# Evaluation of the Removal of Basic Blue 3 and Basic Yellow 28 Textile Dyes from Aqueous Media Using *Persea americana* ‘Hass’ Avocado Peel

**DOI:** 10.3390/molecules31060972

**Published:** 2026-03-13

**Authors:** Türkan Börklü Budak

**Affiliations:** Department of Chemistry, Faculty of Art and Science, Yıldız Technical University, 34220 İstanbul, Türkiye; tborklu@yildiz.edu.tr or turkanborklu@yahoo.com

**Keywords:** adsorption, basic blue 3, basic yellow 28, wastewater, avocado peel

## Abstract

This study investigates the potential of Hass avocado peels as biosorbents for removing the cationic dyes BB3 and BY28 from aqueous solutions. The results demonstrate that agricultural waste can be effectively incorporated into sustainable, environmentally friendly wastewater treatment technologies. The adsorption parameters examined include adsorbent mass (0.1–1.1 g), dye concentration (5–50 mg/L), contact time (5–180 min), stirring speed (100–200 rpm), pH (4–10), and temperature (25–40 °C). UV-Vis spectrophotometric analyses show that the optimum conditions for BY28 removal are 0.3 g/50 mL adsorbent, 5 mg/L dye concentration, 30 min contact time, 200 rpm stirring speed, pH 7, and 40 °C, while those for BB3 are 1.1 g/50 mL adsorbent, 5 mg/L concentration, 45 min contact time, 150 rpm stirring speed, pH 7, and 40 °C. Under the optimized operating conditions, the maximum dye removal efficiencies were 88.24% for BY28 and 99.71% for BB3. This study demonstrates that agricultural waste can be converted into sustainable, reusable biosorbents for removing cationic dyes, thereby contributing to the development of environmentally friendly wastewater treatment technologies.

## 1. Introduction

Cationic synthetic dyes such as Basic Blue 3 and Basic Yellow 28, widely used in textile and related industries, cause serious pollution problems in aquatic environments. Because of their aromatic structures, these dyes resist biological breakdown and, even at low levels, cause notable color changes in water, reducing light penetration. This hampers photosynthesis in aquatic ecosystems and reduces dissolved oxygen levels, negatively affecting aquatic plants and animals [1,2]. However, their discharge without treatment poses a danger to human health, potentially causing skin and eye irritation, as well as many adverse effects that may be toxic and carcinogenic [3]. Basic Yellow 28 is a dye frequently used in the textile industry. In addition to its use at certain doses to maintain aquarium hygiene, its discharge without treatment can create a toxic situation that threatens the natural aquatic environment [1]. These findings emphasize that the presence of synthetic dyes, due to their content of anthraquinones, amines, and ring-like compounds, represents a serious threat to human health and the environment [2]. The data in Table 1 indicate the functional groups that may be present in dye structures, the dye classes to which these groups belong, their properties, and their toxic effects.

Various traditional purification techniques have been explored for treating dye-polluted wastewater, including coagulation–flocculation [5], pressure-driven membrane systems (UF, NF, and RO) [6], ion exchange processes [7], and advanced oxidation methods such as ozonation [8], Fenton-based reactions [9], and UV/H_2_O_2_ systems [10]. Despite their efficacy, these physicochemical methods often necessitate substantial chemical and energy inputs, rely on complex operational setups, and may produce hazardous sludge or secondary pollutants, thereby complicating disposal [11]. Furthermore, their elevated operating costs and the production of significant quantities of treatment residues exacerbate the overall environmental impact [12]. In this context, adsorption emerges as an efficient, environmentally friendly, and cost-effective alternative. Numerous studies have emphasized its benefits, including low operational costs, ease of application, high removal efficiency, and broad applicability across various dye categories [13].

Given these advantages, research on removing dyes from aqueous solutions using agricultural waste as adsorbents is increasing [14]. For example, banana peel was used as an adsorbent, achieving a removal efficiency of 95% (28.8 mg/g) for reactive dye [15]. Similarly, orange peels were used to remove 92.4% (18.6 mg/g) of malachite green dye [16].

In this study, *Persea americana* ‘Hass’ avocado peel has recently gained attention as a renewable biomaterial. Its fibrous structure, mechanical strength, and high water retention capacity indicate its potential to significantly contribute to sustainability [17]. Research has demonstrated that raw and carbonized avocado peels can achieve dye removal rates of 90% and 80% for methylene blue and rhodamine B, respectively [18]. Additionally, a Fe_2_O_4_-BC nanocomposite combined with avocado peel was utilized to remove methylene blue (MB) with an efficiency of 99.9% (60.1 mg/g) [19]. Furthermore, avocado kernel served as an adsorbent for the removal of alizarin red S (ARS) dye, achieving an approximate removal rate of 99.91% (213.6 mg/g) [20]. In another investigation, avocado peel and seed were individually evaluated as adsorbents, successfully removing rhodamine B dye at rates of 88% (13.12 mg/g) and 92% (17.9 mg/g), respectively [21]. As an alternative biosorbent, activated carbon derived from apricot kernels attained removal efficiencies of 87–98% for methylene blue (MB) and 98% for methyl orange (MO) [22]. Moreover, an adsorbent derived from sugarcane was employed to remove BB3 at rates ranging from 16.59% to 42.49% and reactive orange 16 (RO16) at removal rates between 70.48% and 83.33% [23]. Additionally, a molecularly imprinted polymer (MIP), synthesized using a synthetic polymer, achieved a BB3 removal rate of 96.6% (75.12 mg/g) [24].

An examination of the aforementioned studies reveals that there are notable advantages to utilizing avocado peel as a biosorbent within the scope of the present study. For instance, the necessity for chemical modification is apparent when employing similar adsorbents [19,20]. In different applications, thermal pre-activation and preparatory procedures are required [22,23]. The current investigation highlights the practicality and cost-effectiveness of the adsorbent, which is simply prepared through drying and grinding, thus eliminating the need for complex pre-treatment. Additionally, avocado peel is produced abundantly as an industrial byproduct [20,21], representing a waste material that can be repurposed at minimal cost. Its lignocellulosic composition endows it with numerous carboxyl and hydroxyl groups, facilitating interactions with dye molecules via electrostatic attraction, hydrogen bonding, and π–π interactions.

In the present study, considering the aforementioned advantages, avocado peel, which is abundantly available as an agricultural and industrial waste product, was evaluated as a biosorbent, and its performance in removing toxic dyes from wastewater in a practical and low-cost manner was investigated.

## 2. Result and Discussion

### 2.1. Elucidation of the Persea americana ‘Hass’ Avocado Peel

Analyses were conducted to elucidate the structure of the *Persea americana* ‘Hass’ avocado peel. Total protein content was determined by using the Kjeldahl method according to ISO 1871 [25,26], and the results are presented as dry-weight values. Total lipid content was determined according to ISO 12966-2 and ISO 12966-4 standards [27,28]. Fatty acid composition, including polyunsaturated fatty acids (PUFAs), saturated fatty acids (SFAs), monounsaturated fatty acids (MUFAs), and trans fatty acids, was analyzed by gas chromatography (GC). The carbohydrate content of the samples was determined by using the ‘calculation by difference’ method, which involves subtracting other major components from the total mass. In this procedure, the amounts of protein, total lipid (fat), moisture, and ash that make up the sample’s composition were determined using standard analyses; the total weight of these components was then subtracted from the overall weight to express the carbohydrate content, following FAO guidelines [29]. Total lipid (crude fat) content was determined according to ISO 15885 [30] using acid hydrolysis followed by Soxhlet solvent extraction. The ash content of the samples was determined according to ISO 749 [31], in which the samples were incinerated at a controlled high temperature to remove organic matter, and the resulting inorganic residue was weighed. Moisture content was determined according to ISO 665 [32] by drying the sample in an oven until constant mass was achieved. The results obtained are listed in Table 2.

Analysis results show that avocado peels, generally considered waste products, have a high carbohydrate content. In this context, the surface structure contains lignocellulosic functional groups rich in hydroxyl groups, which support the adsorption of cationic dyes, form hydrogen bonds, and enable electrostatic interactions. Furthermore, the presence of both single and multiple unsaturated fatty acids may enhance π–π interactions, thereby promoting adsorption by indicating a hydrophobic biomass surface. These components and similar elements exhibit qualities that underpin the potential of avocado peel as a sustainable biosorbent for dye removal.

### 2.2. The Knowledge of Adsorbent Characterization

#### 2.2.1. SEM Analysis Results

**Figure 1:**
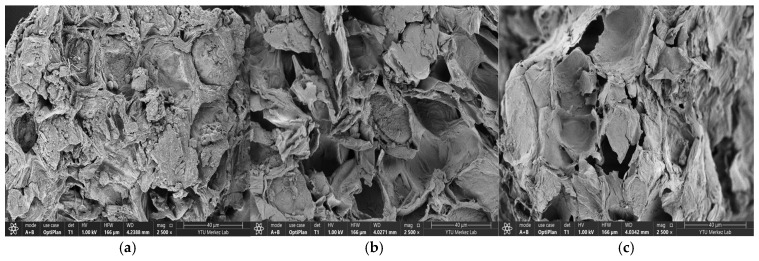
SEM images of *Persea americana* ‘Hass’ avocado peel before adsorption are shown in (**a**), and images after adsorption of BB3 and BY28 dyes are shown in (**b**) and (**c**), respectively.

Studies were conducted to elucidate the morphological properties of *Persea americana* ‘Hass’ avocado peel to enable its use as an adsorbent. A field-emission scanning electron microscope (FE-SEM) was used to analyze surface features. Figure 1a (2500× magnification, HV 1.0 kV, WD 4.2388 mm, and particle size 40 µm) shows the adsorbent obtained from avocado peel before adsorption. In contrast, Figure 1b (2500× magnification, HV 1.0 kV, WD 4.0271 mm, and particle size 40 µm) and Figure 1c (2500× magnification, HV 1.0 kV, WD 4.0342 mm, and particle size 40 µm) show the surface after treatment with BB3 and BY28 dyes, respectively. As shown in Figure 1a, SEM images of the avocado peel adsorbent prior to adsorption reveal a porous and amorphous structure with fibrous, heterogeneous properties and voids of various sizes. This morphologically irregular structure and cracks may offer a propensity to form active and favorable areas for adsorption [33]. In the post-adsorption SEM images shown in Figure 1b,c, it is thought that the surface may have increased in the form of particle layering, possibly due to the coating of the adsorbate surface with dye [34]. In addition to the SEM image data, the absence of new characteristic peaks after adsorption or the lack of a noticeable shift in the peaks in the FT-IR results suggests that the formation of strong chemical bonds during adsorption may have been limited.

#### 2.2.2. FT-IR Measurement Results

**Figure 2:**
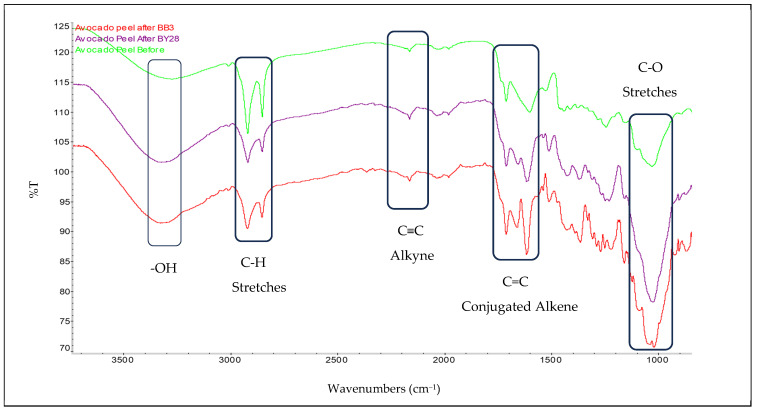
FT-IR spectra before and after the adsorption of avocado peel powder.

Fourier transform infrared spectroscopy (FT-IR) was used to characterize the surface functionalities of the avocado peel-based adsorbent and to identify possible active chemical groups involved in adsorption. The spectrum was collected in the wavenumber range of 4000–500 cm^−1^, and the resulting FT-IR spectrum is shown in Figure 2.

FT-IR spectra of the structures before and after adsorption are shown in Figure 2. The planned band positions in these spectra remained largely unchanged, but differences were observed in the dispersion and width values. After adsorption of the BB3 and BY28 dyes, the spectra in the 3600–3200 cm^−1^ region become broader and denser, making the -OH groups more prominent. This suggests that hydrogen bonds formed between the dye molecules and the avocado adsorbents may be responsible for the change in these bands.

Additionally, aliphatic C–H stretching bands are observed in the spectrum at 2920–2850 cm^−1^. The increase in the distribution of these bands suggests that it may be due to radiation-induced and aliphatic fragmentation of the adsorbent within the dye molecules. The C≡C alkyne band, located around 2100 cm^−1^, is significantly strengthened after adsorption, indicating that these fragmented adsorbent fragments, which may be present in the dye molecules, are bound to the adsorbent.

The sharp, intense C=C bands in the 1600–1500 cm^−1^ range suggest that the dyes’ aromatic structures can adapt to the adsorbent during adsorption. However, the increasing number of bands in the 1300–1000 cm^−1^ region, along with the C–N and C–O expansion rates, supports the possibility of functional interactions between the adsorbent and the dyes used.

The observed spectral changes indicate that interaction between the adsorbent and the dye molecules does not cause structural changes; rather, the dye molecules bind to the adsorbent via surface adhesion. The FT-IR spectra support the idea that adsorption occurs predominantly through physical interactions, with increasing surface density [35,36], as shown by the SEM images of the pre- and post-adsorption structures in Figure 1.

#### 2.2.3. XRD Measurement of Adsorbent Material

**Figure 3:**
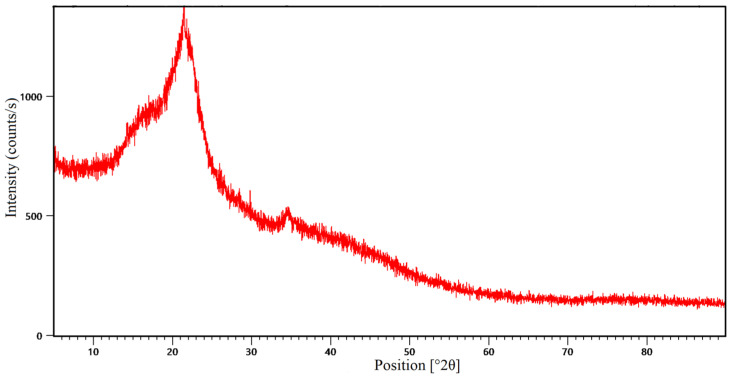
XRD data of avocado peel adsorbent.

The XRD spectra of avocado peel adsorbent powder were evaluated as shown in Figure 3.

As shown in Figure 3, the presence of low-intensity, broad diffraction bands at 2θ ≈ 19.19°, 21.46°, and 22.54° in the XRD pattern, along with the absence of sharp peaks, indicates that the structure is mainly or semi-amorphous (XRD source) [37]. The fact that lignocellulosic biomass-based materials commonly appear in the 2θ ≈ 15–25° range, as reported in the literature [38,39], also supports this association.

#### 2.2.4. BET Analysis of Avocado Peel Adsorbent

**Figure 4:**
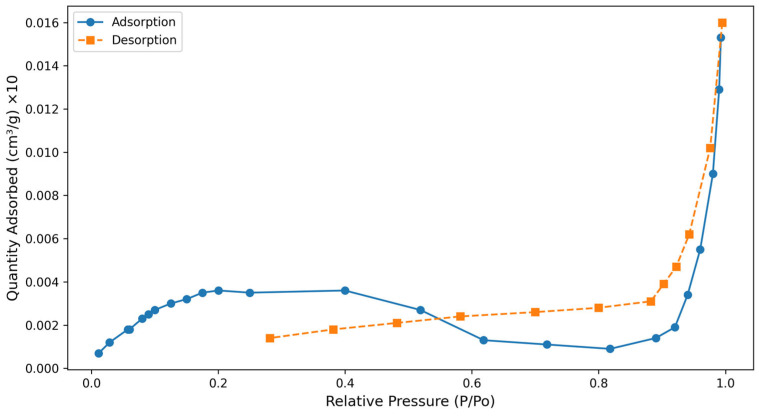
BET results of avocado peel.

The textural properties of the avocado peel adsorbent were characterized using BET measurements. The adsorption and desorption curves in Figure 4 indicate Type IV hysteresis [40]. The pore volume was 0.007 cm^3^/g, the surface area was 0.37 m^2^/g, and the pore width was 7.38 nm. According to the IUPAC classification, the pores were categorized as ultramicropores (<0.7 nm), micropores (0.7–2 nm), mesopores (2–50 nm), and macropores (>50 nm) [41]. In this case, the possibility that the mesoporous structure of the adsorbent may be dominant comes to the fore.

The observed lack of closure in adsorption–desorption isotherms suggests a possible connection to the hysteresis behavior arising from the mesoporous structure of the adsorbed material [42]. In this case, the possibility that complete closure during desorption cannot be achieved due to capillary condensation and the pore structure present in mesoporous materials comes to the fore.

#### 2.2.5. Determination of pH Zero Point Charge

**Figure 5:**
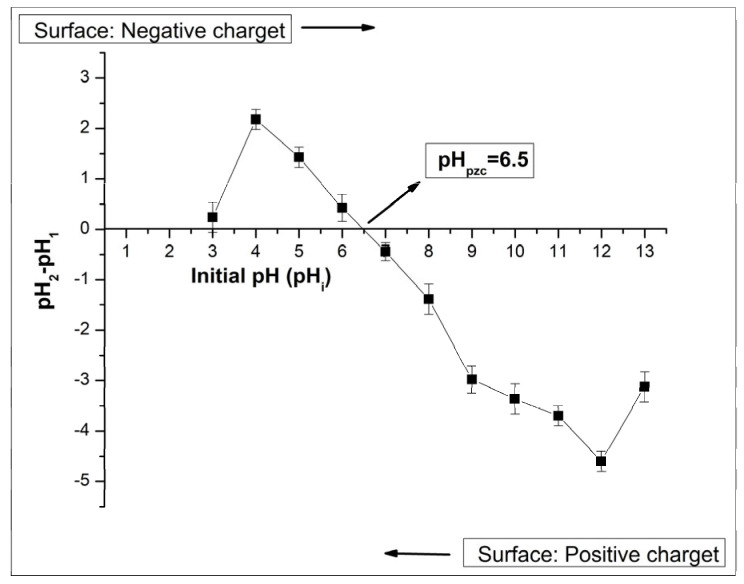
Determination of pHpzc using the pH drift method and the arrow indicates the critical pH value (pH_pzc_ = 6.5).

To determine the adsorbent’s pH zero point charge (pH_pzc_), the pH critical (ΔpH–pH) method, frequently used in the literature, was employed [43,44]. This enabled correlation of removal tendencies with the medium pH during adsorption experiments. Although other complementary approaches, also known as salt addition methods, exist, the pH critical method was deemed sufficient for the purpose and scope of this study. The variation in ΔpH as a function of pH_İ_ was plotted, and the pH_pzc_ was identified as the intersection point of the curve at 6.5 (Figure 5).

The zero-charge point (pHₚzc), the pH at which the biosorbent surface charge is zero, was found to be 6.5 in this study. Therefore, when pH < 6.5, the adsorbent surface will be mostly positively charged, and a decrease in the adsorption of cationic dyes is expected due to electrostatic repulsion [45]. When pH > 6.5, the adsorbent surface will acquire a negative charge, which can enhance its ability to adsorb cationic dyes [46]. It is predicted that the removal percentages of the cationic dyes BB3 and BY28 included in the study may increase under working conditions with pH > 6.5. Indeed, the optimum pH of 7 obtained in the pH optimization study is consistent with this expectation.

#### 2.2.6. UV–Vis Measurements

Dye concentrations were determined by UV-Vis spectrophotometry at λmax = 654 nm for BB3 and 438 nm for BY28, using calibration curves generated from standard solutions and specified in Table 3.

### 2.3. The Results of Adsorption Experiments

#### 2.3.1. Impact of Adsorbent Dosage on Adsorption of BB3 and BY28 Dyes

All experimental studies were conducted with triple parallel replication (*n* = 3), and statistical significance was assessed at the 95% confidence level (*p* < 0.05). To determine the optimal adsorbent amount, one of the important variables affecting adsorption and removal efficiency, experiments were conducted using adsorbent amounts ranging from 0.1 g to 1.1 g. At higher adsorbent concentrations, avocado peel has been observed to cause turbidity and particle clumping in the solution due to its high lipid content.

Since Hass avocado peel has a distinctive color, blank experiments were performed under the same conditions (50 mL distilled water, 25 °C, 60 min, 150 rpm) using the same amount of adsorbent to eliminate background interference. This solution was used as the blank.

**Figure 6:**
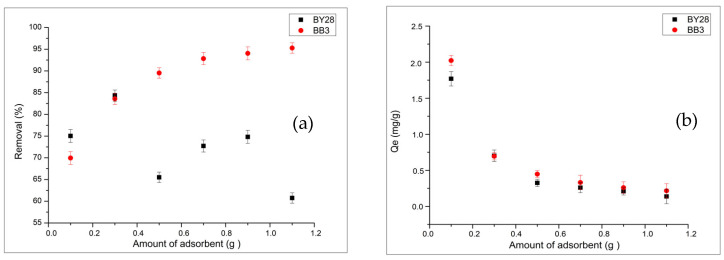
Dependence of BY28 and BB3 removal (**a**) and Qe value (**b**) on adsorbent dosage (Co = 30 mg/L, V = 50 mL, pH 7, 150 rpm, 25 °C, 60 min).

The removal efficiency (%) and adsorption capacity (Qe) for BB3 and BY28 were determined as shown in Figure 6a,b. The highest dye removal was achieved with 1.1 g of avocado peel, yielding 95.28% efficiency for BB3, and with 0.3 g of avocado peel, yielding 84.32% efficiency for BY28. For BB3, the removal rate increases with the amount of adsorbent. This is believed to be due to the adsorbent’s surface binding sites, which are morphologically suitable for binding BB3 molecules, thereby increasing the removal rate. Removal occurs through electrostatic effects. For BY28, removal is supported only up to a certain amount of adsorbent. However, using more than the optimal amount can hinder BY28 binding and may even cause desorption. Van der Waals forces, or π-π interactions, may mediate the high molecular weight of BY28 and its binding. Therefore, properties that promote desorption in the presence of a dense adsorbent may have a more active effect.

#### 2.3.2. Effect of Initial BY28 and BB3 Dye Concentrations of Solution on Adsorption

To assess how initial dye concentration affects adsorption, dye solutions at 5–50 mg/L were prepared, as this is a key factor influencing uptake efficiency.

**Figure 7:**
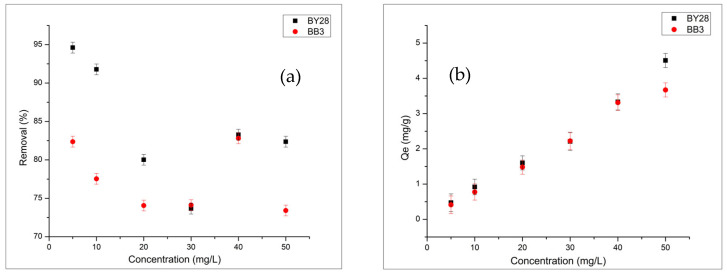
Effect of initial dye concentration on removal (**a**) and Qe (**b**) of BY28 and BB3 (1.1 g adsorbent for BB3 and 0.3 g for BY28, V = 50 mL, pH 7, 150 rpm, 25 °C, 60 min).

As shown in Figure 7a,b the highest removal efficiencies occurred at an initial concentration of 5 mg/L, with 82.37% for BB3 and 94.61% for BY28. The data indicate that the percentage removal rate decreases for both dyes as the initial dye concentration increases, while the adsorbent mass remains constant. This occurs because the adsorbent has a limited number of active sites. Additionally, as the dye concentration increases, the removal percentage drops due to active-site saturation. The rise in Qe, the amount of dye adsorbed per unit of adsorbent, shown in the graphs supports this. Furthermore, the decrease in removal efficiency is likely due to mass transfer limitations at high dye concentrations, intermolecular aggregation, and changes in the ionic environment.

#### 2.3.3. Influence of Contact Time on Adsorption of BB3 and BY28

**Figure 8:**
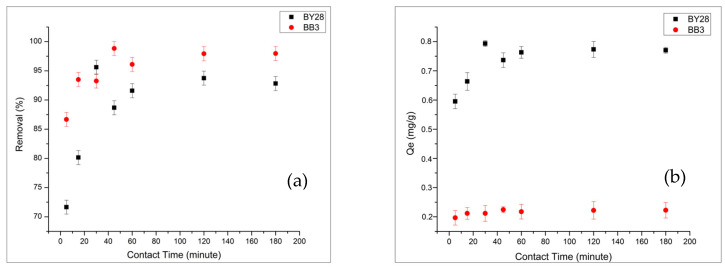
Dependence of BY28 and BB3 removal (**a**) and Qe (**b**) on contact time (1.1 g adsorbent for BB3 and 0.3 g for BY28, V = 50 mL, pH 7, 25 °C, 5 mg/L, and 150 rpm).

To investigate the effect of contact time on adsorption capacity, contact times ranging from 5 to 180 min were used. The results are shown in Figure 8a,b.

As a result, the optimal contact times are 45 min for the BB3 dye and 30 min for the BY28 dye. The adsorption process initially accelerates, significantly contributing to reaching equilibrium, but then slows, making a smaller contribution to the overall equilibrium. This deceleration arises from the limited availability of active sites on the adsorbent surface, which become occupied by dye molecules, reducing the effectiveness of further adsorption. At this point, equilibrium is dynamically achieved between dye molecules adsorbed and desorbed from the adsorbent. This stage is called the “equilibrium time,” and the amount of dye at this stage indicates the adsorbent’s maximum adsorption capacity.

#### 2.3.4. Influence of Agitation Intensity (rpm) on Adsorption of BB3 and BY28

**Figure 9:**
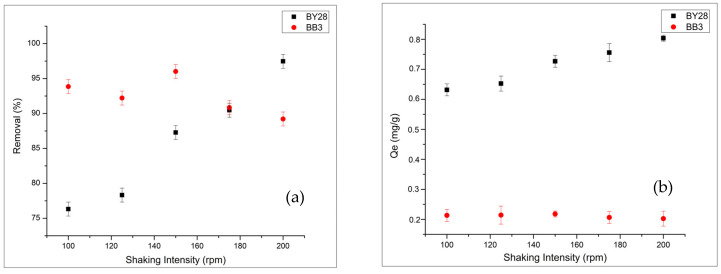
Effect of shaking intensity on removal (**a**) and Qe (**b**) of BY28 and BB3 (1.1 g adsorbent, 45 min for BB3 and 0.3 g, 30 min for BY28. Other conditions: V = 50 mL, pH 7, 25 °C, and 5 mg/L).

To evaluate the effect of shaking intensity, an important parameter affecting adsorption efficiency, shaking intensities were varied from 100 to 200 rpm, as shown in Figure 9a,b. At higher rpm values, the operating range was limited by mechanical instabilities, such as suspension instabilities, and by the negative impact on the mechanical form of the adsorbent.

The results showed that optimal adsorption occurred at 150 rpm for BB3 and at 200 rpm for BY28. At low agitation speeds, the thick diffusion layer in the solution hinders adsorption efficiency from reaching its peak. Conversely, when agitation speeds exceed the optimal level, % removal declines due to increased particle collisions and turbulence.

#### 2.3.5. Influence of Solution Temperature on BB3 and BY28 Adsorption

To examine how temperature affects adsorption behavior and overall efficiency, batch experiments were conducted at 25–40 °C. Temperatures above 40 °C increase energy requirements, limiting practical applications and keeping operations within this range.

**Figure 10:**
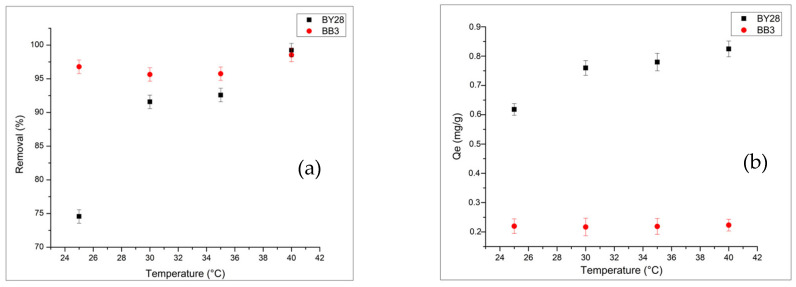
Effect of temperature on removal (**a**) and Qe (**b**) of BY28 and BB3. For BB3, 1.1 g of adsorbent, 45 min, and 150 rpm. For BY28, 0.3 g of adsorbent, 30 min, and 200 rpm. Other conditions: 5 mg/L, 50 mL, and pH 7.

As shown in Figure 10a,b the adsorption performance reaches its highest value at 40 °C within the examined temperature range. This temperature is consistent with Table 4, which presents the thermodynamic analysis results, supporting the conclusion that ΔG° values become more negative at higher temperatures and that the adsorption process is endothermic.

#### 2.3.6. pH Effect on BB3 and BY28 Adsorption

While experimental studies are being conducted to examine the effect of pH on adsorption within the pH range of 4–10.

**Figure 11:**
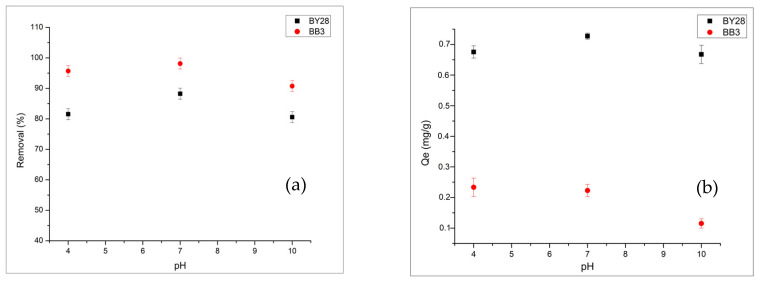
Effect of pH on removal (**a**) and Qe (**b**) of BY28 and BB3. For BB3, 1.1 g of adsorbent, 45 min, and 150 rpm. For BY28, 0.3 g of adsorbent, 30 min, and 200 rpm. Other conditions: 5 mg/L, 50 mL, and 40 °C.

As shown in Figure 11a,b, the highest removal of both cationic dyes occurs at pH 7 (99.71% for BB3, 88.24% for BY28). Although this value, which is above pHpzc 6.5, is expected to provide the highest removal, a similar situation is not observed at pH 10. Literature data indicate that adsorption of cationic dyes at very high pH values is not always favorable [47]. At alkaline pH values ≥ 9–10, the high concentration of OH^−^ ions in the solution can compete with the dye molecules, thereby limiting adsorption efficiency. In addition, studies in the literature indicate that the structural stability and surface-interaction geometries of some cationic dyes can be adversely affected at high pH [48,49]. For these and similar reasons, it is understood that in the moderately alkaline pH range (approximately pH 7–8), where electrostatic attraction is effective, secondary effects are not yet strongly at play, and a more stable adsorption environment is available.

Under the experimental conditions studied, approximately 99.71% of BB3 and 88.24% of BY28 was removed using avocado peel biosorbent. The difference in removal efficiency between the two dyes is thought to be due to differences in the functional groups present in their structures. Accordingly, it is believed that the aromatic triarylmethane structure of BB3 enables stronger π–π interactions with the lignocellulosic components on the biosorbent surface, whereas the azo groups of BY28 may reduce adsorption efficiency due to steric effects and differences in hydration behavior.

#### 2.3.7. Reusability

The reusability of the biosorbent was evaluated separately for each dye through batch-mode experiments, consistent with the procedure used in previous tests. Surface regeneration was investigated by treating the spent biosorbent with 0.1 M HCl, 0.1 M CH_3_COOH, and 0.1 M NaOH solutions.

**Figure 12:**
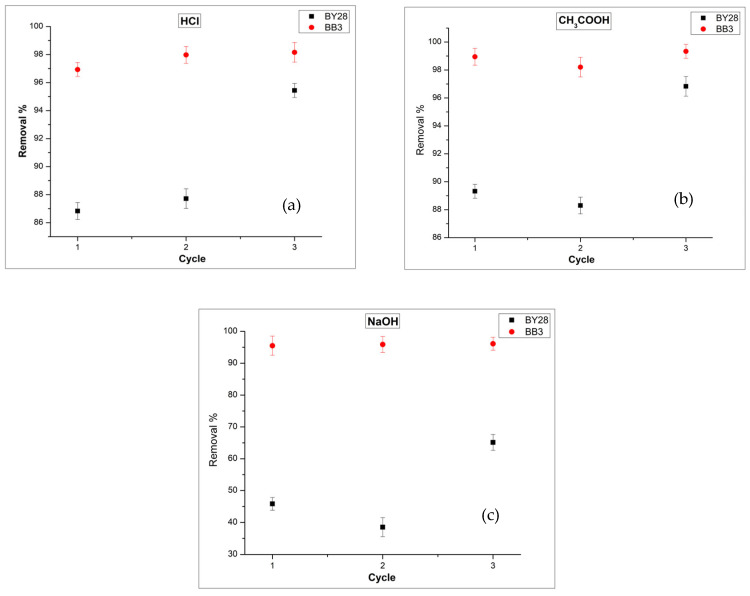
Investigation of the reusability performance of the adsorbent over three adsorption and desorption cycles: 0.1 M HCl (**a**), 0.1 M CH_3_COOH (**b**), and 0.1 M NaOH (**c**). For BB3, 1.1 g of adsorbent, 45 min, and 150 rpm. For BY28, 0.3 g of adsorbent, 30 min, and 200 rpm. Other conditions: 5 mg/L, 50 mL, 40 °C.

Upon examining the data in Figure 12, it is evident that, even after the third re-generation cycle using 0.1 M HCl (a), 0.1 M CH_3_COOH (b), and 0.1 M NaOH (c) eluent solutions, the adsorbent continues to demonstrate high removal efficiency (91.8% for BY28 and 99.3% for BB3). To assess the reusability of the avocado peel biosorbent, three consecutive adsorption–desorption cycles were conducted. After each adsorption, the dye-laden adsorbent was separated and regenerated with an acidic or basic eluent. Subsequently, the adsorbent was rinsed with distilled water, dried, and reused in the next adsorption cycle. This procedure was repeated three times to evaluate the bio-sorbent’s stability and reusability. Maintaining removal efficiency after the third adsorption–desorption cycle suggests that the active sites may have become more accessible due to acid/base treatment, that the structural integrity of the adsorbent may have been preserved, and that its surface properties may have been partially improved [50].

However, the slight change in percentage removal values across repeated cycles is thought to be explained by partial blockage of active adsorption sites and incomplete dye desorption. Acidic regeneration weakens electrostatic interactions by protonating oxygen-containing functional groups such as –OH and C=O on the surface, as seen in the FT-IR spectra in Figure 2. In a basic environment, desorption is thought to be facilitated by electrostatic repulsion and the breaking of hydrogen bonds [51,52]. Negative ΔG° values obtained at 40 °C indicate that adsorption is spontaneous, while positive ΔH° and ΔS° values suggest that the process is endothermic and follows a mechanism supported by an increase in entropy (see Table 4). Such thermodynamic properties are generally associated with reversible, predominantly physical adsorption systems, explaining the high regeneration efficiency. In conclusion, avocado peel powder exhibits good reusability and structural stability and has the potential to be a sustainable wastewater treatment adsorbent [51].

#### 2.3.8. Thermodynamic Evaluation

The thermodynamic parameters, namely Gibbs free energy change (ΔG°), enthalpy change (ΔH°), and entropy change (ΔS°), were calculated using Van’t Hoff plots, which are commonly used in adsorption studies to assess whether adsorption processes are spontaneous. The results are shown in Table 4.

According to these data, both dyes exhibit temperature-dependent behavior. The ΔG° values for dye BB3 approach zero in the temperature range of 25–35 °C (298–313 K), indicating that the adsorption process is near equilibrium at these temperatures. At 40 °C (323 K), a more negative ΔG° is observed, indicating that this temperature favors adsorption. By contrast, the ΔG° value for dye BY28 turns negative with increasing temperature, indicating that adsorption becomes increasingly spontaneous.

The positive ΔH° values for both dyes indicate that adsorption is endothermic [53]. However, as shown in Figure 10, the increase in removal amount with increasing temperature within the examined range suggests that, in addition to physical interactions, temperature-dependent mechanisms may also play a role in the adsorption process. Additionally, the positive ΔS° data indicate that disorder at the solid–liquid interface increases during adsorption and that interactions between the adsorbent and dye molecules become more favorable. These data support the use of avocado peel as an adsorbent for aqueous media containing dyes BB3 and BY28.

#### 2.3.9. Adsorption Isotherms and Statistical Investigation

**Figure 13:**
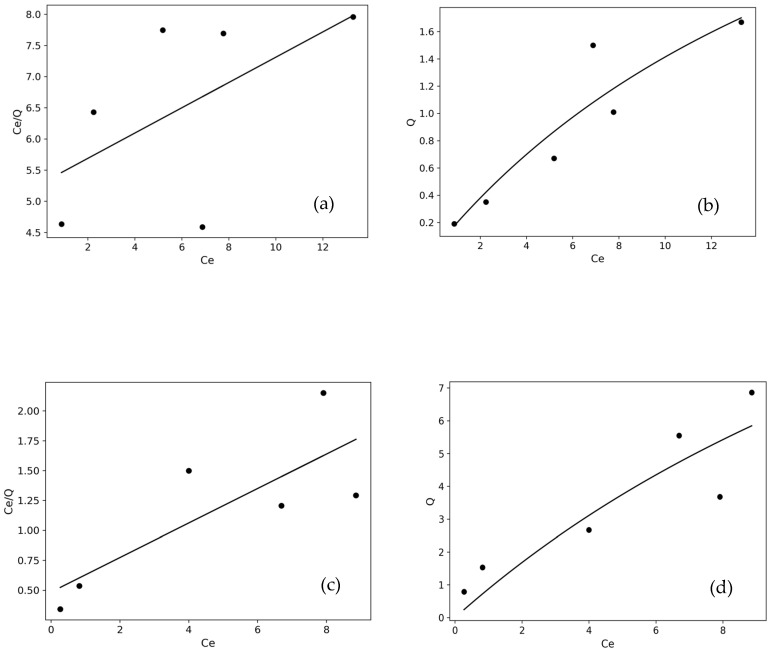
Linear and non-linear Langmuir isotherm plots for BB3 (**a**,**b**) and BY28 (**c**,**d**).

**Figure 14:**
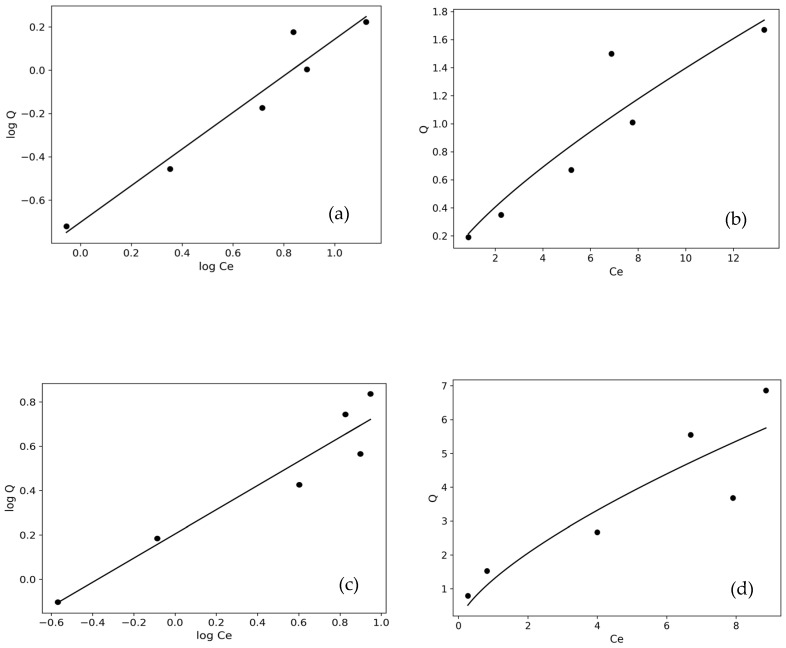
Linear and non-linear Freundlich isotherm plots for BB3 (**a**,**b**) and BY28 (**c**,**d**).

As shown in Figure 13 and Figure 14, analyses were performed using linear and non-linear forms of Langmuir and Freundlich models. The obtained isotherm parameters were evaluated through model fit, R^2^, RMSE, and χ^2^ values [54], and the results are presented in Table 5.

Statistical error analyses (R^2^, RMSE, and χ^2^) for Langmuir and Freundlich isotherm parameter evaluation were not based solely on the correlation coefficient but also considered error functions. For the BY dye, the Qmax value obtained from the non-linear Langmuir model (21.116 mg/g) was significantly higher than that calculated from the linear form (6.936 mg/g). This difference indicates that linearization can alter the error distribution and lead to deviations in parameter estimates. However, when statistical error analyses were considered together, the Freundlich model, which yielded the highest R^2^ and the lowest RMSE and χ^2^ values, was determined to be the most suitable model for BY. For the BB dye, although the non-linear Langmuir model showed an acceptable fit, the Freundlich model performed statistically better, with a higher correlation coefficient and lower error values [23]. In all systems, *n* values greater than 1 (1 < *n* < 10) indicate that the adsorption process is favorable and that a multilayer adsorption mechanism driven by surface heterogeneity may be dominant [36].

#### 2.3.10. Comparative Research

Literature studies on the removal of BB3 and BY28 dyes included in this paper are shown in Table 6. While some adsorbents reported higher removal percentages in the literature (95.22% for BB3 [55]), others were obtained through thermal (99.45% for BB3, 98.01% for BY28 [1]) or ultrasonic-assisted (96.48% [56]) processes. In contrast, the avocado peel adsorbent proposed in this study was prepared by drying and granulation alone, without any chemical or thermal activation. It achieved competitive removal percentages compared with the other adsorbents in the table (99.71% for BB3, 88.24% for BY28).

However, the results obtained should not be considered a direct comparison; they depend on various parameters, including the surface morphology of the adsorbent and the nature of its functional groups; the optimization of experimental conditions; the charge properties of the dye molecules; and the type of adsorbent–dye interactions. Furthermore, this study was conducted at a neutral or slightly alkaline pH (pH ≈ 7) over a relatively short time period, indicating that the proposed adsorbent has promising performance for practical applications.

## 3. Experimental

### 3.1. Materials

Stock solutions of Basic Blue 3 (BB3; C_20_H_26_ClN_3_O, molecular weight 359.89 g/mol, λ_max_ = 654 nm, purchased from Sigma-Aldrich (Merck KGaA, St. Louis, MO, USA), approximately 25% content, used without further purification) and Basic Yellow 28 (BY28; C_16_H_18_N_4_S, molecular weight 433.5 g/mol, λ_max_ = 438 nm, BY28 dye (analytical grade), obtained from Sigma-Aldrich (Merck KGaA, St. Louis, MO, USA), and used as received) were prepared at a concentration of 1000 mg/L. From these stock solutions, working solutions at concentrations of 5–50 mg/L were prepared by serial dilution with distilled water. The adsorbent material used in this study was the Hass variety of avocado, collected from the Gazipaşa area in Antalya, Türkiye.

### 3.2. Instrumentation

All experiments used water from an 8 L Mikrotest MSD-08 (Mikrotest, İstanbul, Türkiye) purification system, which had a conductivity of about 2.3 µS/cm. Heating and drying were performed in a Memmert oven (Memmert GmbH + Co. KG, Schwabach, Germany) at temperatures from +20 °C to +300 °C. The setup included an analytical balance (Radwag), a drying oven (Memmert UN 55, Co. KG, Schwabach, Germany), a water purification unit for distilled water (Mikrotest MSD-08, İstanbul, Türkiye), a UV-Vis spectrophotometer (Agilent 8453, Agilent Technologies, Santa Clara, CA, USA), a thermostatic shaking water bath (Julabo SW 22, ISOLAB Laborgeräte GmbH, Eschau, Germany), and a pH meter (WTW InoLab Cond pH 720, WTW, Weilheim, Germany). For adsorption experiments, a Julabo SW 22 (ISOLAB Laborgeräte GmbH, Eschau, Germany) heated and shaking water bath was used. The change in dye concentration was measured with a UV-Vis spectrophotometer (Agilent 8453, 190–1100 nm, Agilent Technologies, Santa Clara, CA, USA). The functional groups of the adsorbent were analyzed with a Nicolet IS 10 FT-IR spectrometer (FT-IR-ATR; Nicolet IS 10, Thermo Fisher Scientific Inc., Waltham, MA, USA). During measurements, 32 scans were taken at a 4 cm resolution, covering 4000–400 cm^−1^, using an ATR diamond crystal for each measurement. To thoroughly characterize the adsorbent, XRD analysis was performed with a Malvern PANalytical X’ Pert PRO (UK), operating at 40 mA and 45 kV, with a goniometer radius of 240 mm and a focus-divergent slit distance of 144 mm. The Field Emission Scanning Electron Microscope (FE-SEM, Thermo Scientific Apreo 2 S LoVac, Waltham, MA, USA) was used to examine the crystal structure and morphology, with the following settings: 40 µm, HV 0 kV, WD 4.2388 mm, and magnification 2500 KX. The textural properties, including pore volume and specific surface area, were evaluated using the Brunauer–Emmett–Teller (BET) method (Micromeritics ASAP 2020, Norcross, GA, USA). Adsorption properties were determined in nitrogen at −195.441 °C using an analysis bath, with temperature equilibration at 10 s intervals.

### 3.3. Preparation of Avocado Peels

**Figure 15:**
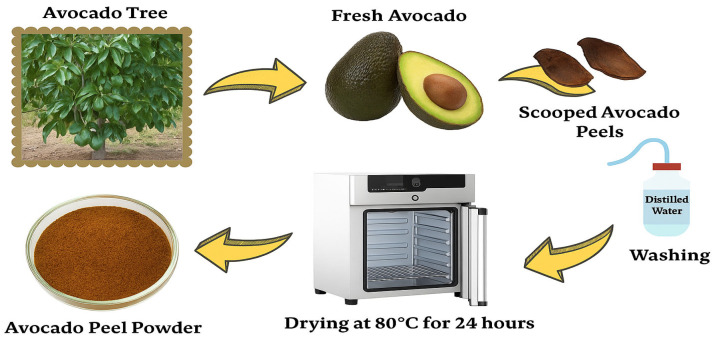
Avocado peels used as an adsorbent.

To remove any contaminants from the avocado peels, they were first washed with tap water and then with distilled water. They were then oven-dried at 80 °C for 24 h (Figure 15). After drying, the shells were broken into small pieces and sifted through a 24-mesh sieve to prepare them for use. Avocados were sourced from Antalya or Gazipaşa, Türkiye.

### 3.4. Adsorption Behavior with BY28 and BB3 Dyes

The batch method was used to conduct adsorption experiments. For this, 50 mL of BY28 and BB3 dye solutions at specific concentrations were prepared from a 1000 mg/L stock solution and transferred into 100 mL glass beakers. Experiments were conducted under laboratory conditions. The effects of various parameters, including adsorbent dose (0.1–1.1 g), initial dye concentration (5–50 mg/L), contact time (5–180 min), shaking water bath speed (100–200 rpm), temperature (25–40 °C), and pH (4–10), were studied. After each experiment, the solutions were filtered through filter paper, and the absorbance was measured at 438 nm for BY28 and at 654 nm for BB3 using a UV-2600 UV-Vis spectrophotometer (Shimadzu, Kyoto, Japan). The removal efficiency (%R) and adsorption capacity (Qe) were calculated using Equations (1) and (2) [62,63].

(1) $$\%R=\frac{{(C}_{o}-C_{e})}{C_{o}}\times 100$$

(2) $$Q_{e}=\frac{{(C}_{o}-C_{e})}{m}\times V$$

Here, *C_o_* is the initial dye concentration (mg/L), *Cₑ* is the equilibrium concentration (mg/L), *m* is the adsorbent mass (g), and *V* is the dye solution volume (L).

### 3.5. Method for Determining pH_pzc_ (Point of Zero Charge)

To determine the point of zero charge (pHpzc), a key parameter influencing adsorption behavior, a series of 11 beakers (50 mL each) containing NaNO_3_ electrolyte solutions was prepared, and the initial pH (pH_1_) was adjusted between 3 and 13. Then, 0.50 g of the adsorbent was added to each beaker, and the mixtures were allowed to reach equilibrium at room temperature for 48 h. Afterward, the final pH (pH_2_) was recorded, and the difference between the final and initial pH (ΔpH = pH_2_ − pH_1_) was calculated. In the graphs, the initial pH (pHi) was plotted on the *x*-axis, and the change in pH (ΔpH = pHf − pHi) on the *y*-axis [64].

### 3.6. Regeneration Procedure

Regeneration experiments were conducted to evaluate the reusability of avocado peels. The primary aim of regeneration is to assess economic viability and to reduce sludge formation as a secondary pollutant [19]. For this purpose, experiments were repeated using 0.1 M HCl, 0.1 M CH_3_COOH, and 0.1 M NaOH eluent solutions in a sequential adsorption–desorption cycle. Previous studies involving adsorbents derived from avocado biomass have also reported that regeneration efficiency can be maintained at high levels across multiple cycles, with only a gradual decline observed [20]. In this study, consistent with existing literature, the adsorption–desorption process was reiterated over three consecutive cycles to assess the stability and regeneration potential of the avocado peel adsorbent.

For this purpose, the adsorption process was conducted by utilizing the parameter values that yielded the highest efficiency among the conditions investigated. The dye molecules accumulated on the adsorbent were subsequently removed by agitating with 50 mL of eluent solution at 150 rpm at ambient temperature for a duration of 2 h. Following this, the mixture was filtered, and the adsorbent was washed with distilled water and subsequently dried at 80 °C for 6 h. To enable reuse as an adsorbent, the optimal operating conditions were established; dye solution was introduced, and the procedure was reiterated three times.

### 3.7. Thermodynamic Analysis

To analyze the adsorption process from a thermodynamic perspective, the Gibbs free energy change (ΔG°), enthalpy change (ΔH°), and entropy change (ΔS°) were calculated. Equations (3)–(5) were used in this process.

(3) $$\Delta G^{\circ }=-RTlnK_{d}$$

(4) $$K_{d}=\frac{Q_{e}}{C_{e}}$$

(5) $$lnK_{d}=-\frac{\Delta H^{\circ }}{R}\left(\frac{1}{T}\right)+\frac{\Delta S^{\circ }}{R}$$

In Equations (3) and (4), R represents the universal gas constant (8.314 J/mol K), T represents the absolute temperature (K), Kd represents the distribution coefficient, Qe (mg/g) represents the amount of dye adsorbed at equilibrium, and Ce (mg/L) represents the equilibrium concentration. For Equation (5), the slope of the plot of ln Kd versus 1/T equals −ΔH°/R, and the ordinate intercept equals ΔS°/R.

### 3.8. Isotherm Modeling and Statistical Evaluation

Adsorption isotherms were used to determine how dye molecules interact with adsorbent surfaces and to evaluate their adsorption capacities [65,66,67]. The Langmuir and Freundlich isotherm models in Equations (6)–(9) shown in Table 7 were applied to the obtained data. In addition, Equations (10)–(12) in Table 7 were used for statistical evaluation.

In these equations, Q_e_ (mg/g) represents the amount of dye adsorbed at equilibrium, *C*_e_ (mg/L) represents the dye concentration at equilibrium, Q_max_ (mg/g) represents the maximum monolayer adsorption capacity, and *K*_*L*_ (L/mg) represents the Langmuir constant related to adsorption energy. *R*_L_ is the dimensionless separation factor used to assess the suitability of adsorption. *K*_F_ represents the adsorption capacity in the Freundlich model, while *n* reflects surface heterogeneity and adsorption density. Q_e__exp and Q_e__calc represent the experimental and model-calculated adsorption capacity values, respectively, and N represents the number of data points. RMSE and χ^2^ values were calculated only for non-linear model fits, as linear transformations can alter the error distribution.

## 4. Conclusions

In this study, avocado peel, a food industry byproduct often considered waste, was used as a low-cost adsorbent to remove the dyes Basic Blue 3 (BB3) and Basic Yellow 28 (BY28) from aqueous media. The study optimized parameters, including adsorbent dosage, initial dye concentration, contact time, dye–adsorbent contact rate, temperature, and pH. The highest removal rate for BB3 was 99.71%, achieved with 1.1 g of adsorbent, a 5 mg/L dye concentration, 45 min of contact time, 150 rpm agitation, 40 °C ambient temperature, and pH 7. When tested for BY28 under the same parameters, an 88.24% removal rate was achieved with 0.3 g of adsorbent, a 5 mg/L dye concentration, 30 min of contact time, 200 rpm shaking, 40 °C ambient temperature, and pH 7.

Furthermore, in thermodynamic investigations, the obtained ΔG°, ΔH°, and ΔS° values suggest that the adsorption process is predominantly favorable from both physical and energetic perspectives. SEM analyses of the post-adsorption structure show increased particle volume and clustering, visually supporting dye retention. Similarly, the fact that the positions of the functional group bands in the examined FT-IR spectra did not change before and after adsorption, while their intensities differed, suggests that the surface–dye interactions were reversible and predominantly physical in nature. The XRD results of the adsorbent reveal a predominantly amorphous structure, indicating favorable surface properties for adsorption. The instrumental analyses performed generally serve as a complementary contribution to the interpretation of experimental and thermodynamic data, rather than definitively proving the adsorption mechanism.

When the reusability of the adsorbent was examined, it was found to effectively remove the dyes even after three adsorption–desorption cycles. Furthermore, the adsorption data indicate that the Freundlich model fit well for both dyes. Adsorption capacities were determined to be 21.116 mg/L for BY28 and 4.928 mg/L for BB3.

Overall, the findings indicate that avocado peel powder has potential as an environmentally friendly, low-cost adsorbent for removing synthetic dyes from aqueous media. This study shows that agricultural waste can serve as a sustainable alternative for wastewater treatment in textile and related industries.

## Figures and Tables

**Table 1:** Classes of dyes, chemical groups, and associated pollution [4,5].

Dye Class	Solubility & Key Features	Dominant Chemical Groups	Pollution-Related Consequences
Acid	Water-soluble; typically anionic dyes applicable to proteins such as wool, silk, nylon; dyed under acidic conditions.	Azo, anthraquinone, triarylmethane	Causes coloration, increases organic acids, and contributes unreacted dye flux into effluent
Basic	Water-soluble; cationic, high-brightness dyes mainly used on acrylic, some wool/silk	Azo, triarylmethane, anthraquinone, oxazine, acridine	Causes intense color, leaves unbound dye in wastewater
Direct	Water-soluble; substantive dyes for cellulose fibers; applied in neutral to slightly alkaline baths, often with salt	Azo, phthalocyanine, stilbene, oxazine	Coloration, residual salts, unbound dye, and surfactants in effluent
Disperse	Water-insoluble; used for hydrophobic synthetic fibers like polyester; applied at high temperatures	Azo, anthraquinone, nitro	Intense color, organic acids, phosphates, defoamers, bleaching agents, dispersants
Reactive	Water-soluble; largest dye class; forms covalent bonds with cellulose under alkaline conditions	Azo, anthraquinone, phthalocyanine, formazan, oxazine	Coloration, high salt content, alkalinity, unbound dye (from hydrolysis of unreacted dye), surfactants, defoamers in effluent

**Table 2:** Chemical profile of *Persea americana* ‘‘Hass’’ avocado peel.

Name of Analysis	Results	Applied Methods	References
Total protein	7.3 g/100 g ± 0.3	ISO 1871	[25,26]
Total amount of polyunsaturated fatty acids	18.7% ± 1.5	ISO 12966-2/4	[27,28]
Saturated fatty acids	21.5% ± 2.2	ISO 12966-2/4	[27,28]
Monounsaturated fatty acids	52.7% ± 1.9	ISO 12966-2/4	[27,28]
Trans fatty acids	<0.5%	ISO 12966-2/4	[27,28]
Carbohydrate content	58.9 g/100 g ± 2.6	FAO	[29]
Total amount of fat	5.1% ± 0.2	ISO 15885	[30]
Ash content	4.2% ± 0.6	ISO 749	[31]
Humidity analysis	7.6% ± 0.4	ISO 665	[32]

**Table 3:** Parameters of the UV-VIS method employed for the determination of BB3 and BY28.

BB3	(654 nm)	Y = 0.0979X + 0.1971	R^2^ = 0.9891
BY28	(438 nm)	Y = 0.0818X − 0.1387	R^2^ = 0.9917

**Table 4:** Thermodynamic parameters for BB3 and BY28 adsorption onto avocado peel adsorbent.

Thermodynamic Parameters			
BB3			
Temperature (K)	∆G° (kJ/mol)	∆H° (kJ/mol)	∆S° (J/mol K)
298	−0.70	+40.05	+133.94
303	+0.12		
313	0.00		
323	−2.97		
BY28			
Temperature (K)	∆G° (kJ/mol)	∆H° (kJ/mol)	∆S° (J/mol K)
298	+1.82	+87.7	+307.00
303	−1.48		
313	−1.88		
323	−7.85		

**Table 5:** Langmuir, Freundlich isotherms, and statistical results for the adsorption of BB3 and BY28 are presented.

Dye	Model	Type	Q_max_ (mg/g)	K_L_ (L/mg)	K_F_	N	R^2^	RMSE (mg/g)	χ^2^
BB3	Langmuir	Linear	4.928	0.038	–	–	0.329	-	-
BB3	Freundlich	Linear	–	–	0.199	1.183	0.941	-	-
BB3	Langmuir	Non-linear	4.453	0.047	–	–	0.862	0.205	0.247
BB3	Freundlich	Non-linear	–	–	0.237	1.299	0.853	0.212	0.273
BY28	Langmuir	Linear	6.936	0.298	–	–	0.639	-	-
BY28	Freundlich	Linear	–	–	1.602	1.835	0.92	-	-
BY28	Langmuir	Non-linear	21.116	0.043	–	–	0.791	0.977	3.045
BY28	Freundlich	Non-linear	–	–	1.271	1.445	0.809	0.935	1.294

**Table 6:** Adsorption capacities of different adsorbents for BB3 and BY28 dyes: comparison based on literature reports.

Dye	Adsorbent/Material	Method/Characteristics	Removal (%)	References
BB3	Hass avocado peel (this study)	Natural, dried, ground	99.71	[This study]
BB3	SLM Stem—activated at 800 °C	Thermal activation	99.45	[1]
BB3	SLM Stem—natural	Physically prepared	84.82	[1]
BB3	Sugarcane bagasse (NSB)	Ground natural material	77.65	[57]
BB3	QSB—Quaternized sugarcane bagasse	Surface modification	16.52	[57]
BB3	PANI/Fe_3_O_4_ nanocomposite	Magnetic polymer composite	71.02	[58]
BB3	Activated carbon (from rattan wood)	Chemical activation	95.22	[55]
BB3	Palm fiber-based activated carbon	Low-cost agricultural waste	98.31	[59]
BY28	Hass avocado peel (this study)	Natural, dried, ground	88.24	[This study]
BY28	Fly ash + soil mixture	Hybrid adsorbent	98.21	[18]
BY28	SLM Stem—activated at 800 °C	Thermal activation	98.01	[1]
BY28	SLM Stem—natural	Natural adsorbent	58.12	[1]
BY28	Hydromagnesite stromatolite	Ultrasonically assisted adsorption	96.48	[56]
BY28	Beneficiated kaolin	Natural, beneficiated kaolin	94.71	[60]
BY28	ZnO/Mg_3_B_2_O_6_ nanocomposite	Sol–gel synthesis at 650 °C	92.51	[2]
BY28	Mulberry leaves	Natural plant-based adsorbent	98.88	[61]

**Table 7:** Isotherm models and statistical equations were used in this study.

Model/Function	Equation	No
Langmuir (Non-linear)	$\begin{array}{c} Q_{e}=\frac{Q_{max}K_{L}C_{e}}{1+K_{L}C_{e}} \end{array}$	(6)
Langmuir (Linear)	$\begin{array}{c} \frac{C_{e}}{Q_{e}}=\frac{1}{K_{L}Q_{max}}+\frac{C_{e}}{Q_{max}} \end{array}$	(7)
Freundlich (Non-linear)	$\begin{array}{cc} & Q_{e}=K_{F}C_{e}^{1/n} \end{array}$	(8)
Freundlich (Linear)	$logQ_{e}=logK_{F}+\frac{1}{n}logC_{e}$	(9)
Coefficient of	$\begin{array}{cc} {\;\;\;\;\;\;\;\;\;\;\;\;R}^{2}=1-\frac{\sum (Q_{e,exp}-Q_{e,calc}{)}^{2}}{\sum (Q_{e,exp}-\bar{Q_{e,exp}}{)}^{2}} & \end{array}$	(10)
Determination (R^2^)		
Root Mean Square Error	$\;\;\begin{array}{ccc} & RMSE=\sqrt{\frac{1}{N}\sum (Q_{e,exp}-Q_{e,calc}{)}^{2}} & \end{array}$	(11)
(RMSE)		
Chi-square ($\chi^{2}$)	$\begin{array}{c} \chi^{2}=\sum \frac{\left(Q_{e,exp}-Q_{e,calc}{)}^{2}\right.}{Q_{e,calc}} \end{array}$	(12)

## Data Availability

The original contributions presented in this study are included in the article. Further inquiries can be directed to the corresponding author.
